# Leeches and How I Manage Them

**Published:** 1874-03

**Authors:** 


### ARTICLE IV.

### *Leeches, and How I Manage Them.*

BY A COUNTRY CHEMIST.

There are two kinds of leeches recognized by dealers—namely, the green leech (*Sanguisuga officinalis*,) and the speckled or brown leech (*S. medicinalis.*) Our native species belongs to the latter. Sometimes it is difficult to detect any characteristic difference between the green and speckled leeches, and by many eminent naturalists they are supposed to be one species. The former is a native of the south of Europe, and is frequently called the "Hungary Leech," from its being extensively exported from that country. The American pharmacists use a very distinct species (*S. decora.*) Our official leeches may be distinguished by one having a spotted yellow belly, the other is not speckled, but of a greenish yellow.

Leeches are not now so much thought of as in the days of our forefathers. This probably is the reason why we find so little knowledge respecting their habits and employment amongst chemists. If they were more frequently called for by customers, so as to become a profitable branch of the trade, their use and habits would be more studied. The leech, as is well known, is employed only to subtract blood in some local part and in small quantities. The narrowest end or part contains the mouth, the broad and flat end is merely a sucker to hold on to the skin.

The mouth is a triangular aperture furnished with from seventy to ninety teeth, very minute certainly, but called teeth by naturalists. By means of these skin is broken whilst a continual swing-like motion is experienced when the creature is sucking up the blood. It is thought the mouth keeps open the wound whilst sucking, but this is only conjectural; probably it acts upon the same principle as a sponge. The physician when ordering his patient to apply leeches, should mark out the exact spot where they are to be placed with pen and ink, for sometimes ignorant persons, especially when applying them to the abdomen, allow them to wander about and suck anywhere, but if the place is marked out they would be more careful. The proverbial impossibility of making a horse drink against its will applies equally to leeches. It is at times difficult to persuade them to bite; even when they are induced it is, perhaps, only for a few seconds, and they wander away again. This may arise from several causes. It may be the blood is so impure that directly it is tasted the leech refuses to suck any more; this, however, is seldom the case. If the skin is at all unclean it is useless to apply them. The chemist who sells the leeches should be careful to inform the applicant of this fact; it will save much annoyance. The part should first be well sponged with warm water, then rubbed with a little milk. Sometimes it is well just to gently prick the part with a fine needle, or to rub a small quantity of blood over the skin. I have also seen milk and sugar used with good effect. In cold weather, before applying leeches, they should be placed in warm water about 75° Fahr.—if a tablespoonful of beer is mixed with the water all the better—then for a few minutes allow them to crawl about over a rough cloth or towel; they will afterwards generally bite very freely.

If they are too lively a good plan is to confine them beneath a wineglass until they have fastened on the skin. Many chemists recommend their customers to purchase leech glasses (tubes;) I have found these useful when they are applied in the mouth, but I prefer to work without the tube when applying them to any other part of the body; a wineglass, which is found in almost every house, is all that is required. Persons unused to them do not know the difference between the head and tail, therefore in using the tubes they are apt to apply the tail end to the skin.

I know it is not an uncommon fraud to sell leeches as virgin or new leeches which have been previously used. Dr. Christison's directions for discovering this deception should be known by everybody. He says "the gorging of leeches is a more common fraud than the substitution of spurious species. They are known by being less velvety in their coat, less flat when pressed, and presenting a little tumor when squeezed betwixt the fingers from the head to the tail. Leeches which have been used are often sold for unused or virgin leeches. They are best known by putting them on a white cloth and dusting their forepart with finely powdered salt; in thirty seconds a little blood will be emitted, but not a particle if the leech be quite fresh."

It is not a pleasant thought to fancy a leech is being applied to your skin, which has been sucking some patient in a fever ward of a hospital.

Of course no reasonable person can deny that leeches, if healthy, can be used more than once; even Christison states he has used them three days in succession without impairing their activity by immersing them in a solution of sugar and water frequently changed. Directly after removing them from the skin they are commonly sprinkled with fine salt on a plate to cause them to disgorge the blood. To me this appears cruel, not only to watch the leech writhing in agony, but to observe the skin wrinkled where the salt has touched. I just cover them with brown or raw sugar for a few minutes; afterwards it is well to pass them betwixt the finger and thumb holding it by the tail, then place them in water in which a very small quantity of sugar has been dissolved, and change often for the first week. Workingmen and others, to whom the expense of leeches is a consideration, should be instructed as above by the chemist when giving him the leeches; they may possibly be required again by his medical attendant; if so it will save him much expense. A good mode of testing the healthiness of leeches is to hold them for a minute or so in the palm of the hand. The best and healthiest specimens will immediately contract into a roundish ball-like form; these seldom fail to give satisfaction.

Frequently it is desirable to draw more blood after removing the leech. To do so, hot flannels or linen rags heated and held on the part answer admirably. I have invariably found this the most satisfactory method, far more so than applying hot bran or bread poultices. One thing should not be overlooked, the difficulty in stopping the flow of blood. This seems never to enter the minds of most individuals, yet there are patients met with in every practitioner's experience in whom it is exceedingly difficult to stop bleeding. If a case like this should be suspected when applying the leech, place it if possible and convenient over a bone, where pressure from the finger will often stop the bleeding. If this will not succeed, moistened matico leaves are useful, or linen rags soaked in a strong solution of alum. When the patient is strong and not suffering from any debilitating illness, the loss of a little blood will not be felt, but in the case of a weak child, or a person reduced by disease, it is often important to stay the flow of blood immediately, or they might be so weakened by its loss as to cause fatal results.

Now a word on keeping leeches. I have heard many of my drug friends complain sorely about the loss from this source. To preserve them without loss and in a healthy state we should strive as closely as possible to imitate nature. How can they be expected to live in a fancy glass jar, exposed in a window, beneath the heat of a summer's sun? When I first entered into business, I ordered from a London firm, a hundred speckled Hambro' leeches, which I placed in a large glass globe, but although they were apparently healthy when received, they soon sickened and died. I do not think I sold a single dozen out of the whole lot. I thought this sort of thing would never pay, so I procured a fancy jar, or aquarium, made on purpose, and advertised extensively. It was to be stocked with *Valisneria spiralis* and other water weeds, water snails, sand at the bottom, etc., but I was still far from successful. At last I became so disheartened that I gave up keeping leeches altogether. I had no sooner so resolved, then naturally the demand increased, and in deference to the wishes of a medical practitioner in the neighborhood, I decided to give the leech trade one more trial. I purchased a large black earthenware jar, with a wide mouth, glazed inside, such as is used by frugal houswives as a pickle jar, only mine will hold nearly two gallons of water. I place at the bottom a layer an inch in thickness, of large pebbles and sand well washed, then I fill the jar about two-thirds with water, hard or spring water does not answer so well as rain water. I change the water on an average every fortnight in the summer in the winter season once a month is sufficient, for then the leeches are torpid; I never allow the water to freeze although it frequently reaches as low as 40° Fahr. The jar is kept in a cool place. I now seldom loose any of my leeches, if the consignment is healthy when received.

I believe the chief cause of failure is exposing them too freely to the light, which excites them. In their native habitat, they live for the greater part of their lives beneath the mud at the bottom of the lakes, where they live in a semi-torpid state; in a dark jar, or where the light is kept from them, they are also semi-torpid.—*Chemist and Druggist.*

